# Functional changes in prefrontal cortex following frequency-specific training

**DOI:** 10.1038/s41598-022-24088-7

**Published:** 2022-11-24

**Authors:** Lana Bach-Morrow, Francesco Boccalatte, Antonio DeRosa, David Devos, Carmen Garcia-Sanchez, Matilde Inglese, Amgad Droby

**Affiliations:** 1 Think Interfaces, New York, NY USA; 2 Department of Pathology, NYU Langone Medical Center, New York, NY USA; 3 Department of Mathematics, University of Maryland, College Park, MD USA; 4 Department of Neurology, University Hospital, Univ of Lille, Lille, France; 5 Neuropsychology Unit, Neurology Service, Hospital de Sant Pau, Barcelona, Spain; 6 Neurology Department, Icahn School of Medicine at Mount Sinai, New York, NY USA

**Keywords:** Neural circuits, Imaging techniques, Biotechnology, Neuroscience, Medical research, Neurology

## Abstract

Numerous studies indicate a significant role of pre-frontal circuits (PFC) connectivity involving attentional and reward neural networks within attention deficit hyperactivity disorder (ADHD) pathophysiology. To date, the neural mechanisms underlying the utility of non-invasive frequency-specific training systems in ADHD remediation remain underexplored. To address this issue, we created a portable electroencephalography (EEG)-based wireless system consisting of a novel headset, electrodes, and neuro program, named frequency specific cognitive training (FSCT). In a double-blind, randomized, controlled study we investigated the training effects in N = 46 school-age children ages 6–18 years with ADHD. 23 children in experimental group who underwent FCST training showed an increase in scholastic performance and meliorated their performance on neuropsychological tests associated with executive functions and memory. Their results were compared to 23 age-matched participants who underwent training with placebo (pFSCT). Electroencephalogram (EEG) data collected from participants trained with FSCT showed a significant increase in 14–18 Hz EEG frequencies in PFC brain regions, activities that indicated brain activation in frontal brain regions, the caudate nucleus, and putamen. These results demonstrate that FSCT targets specific prefrontal and striatal areas in children with ADHD, suggesting a beneficial modality for non-invasive modulation of brain areas implicated in attention and executive functions.

## Introduction

The prevalence of children diagnosed with attentional issues and attention deficit hyperactivity disorder (ADHD) is increasing both in the US and globally. Markers of ADHD are, amongst others, inattentiveness, lack of focus, inability to self-sustain attention for a prolonged amount of time, and decrease in working memory^1^.

Previous studies indicate that specific loci within the prefrontal cortex (PFC) play a significant role in a variety of attentional functions. Numerous studies reported associations between specific EEG frequencies and activation in these PFC regions^2,3^. In their recent study, Bedini and Baldauf demonstrated that along with topographic organization, the structure, function, and connectivity define the concept of a cortical region^4,5^. Previous studies already elaborated on the hypothesis that PFC can be segregated into functionally distinct domains^6^, given substantial differences in the selectivity of neurons^7^, as well as their anatomical connectivity patterns^6^.

The underlying circuits within PFC, (as well as their oscillations and respective connectivity patterns) sub-serve the attentional functions, and several of these circuits are directly related to the development of ADHD. One area of particular interest is the inferior frontal junction (IFJ)^4,8^, which is implicated in a cognitive control network^9^. Recently, the IFJ has sparked additional interest given its involvement in multiple high-level cognitive functions, such as top-down visual attention^10^, working memory^11^, and the implementation of novel task instructions. Furthermore, neurons projecting from the brainstem and basal forebrain areas to the nucleus accumbens, hippocampus, and amygdala, are also regulated by PFC projections and seem to be of relevance in modulating motor, emotional, and memory functions. Both physiological and pathological changes in the PFC influence the activity of these areas and the corresponding goal-oriented behaviors^12^.

Previous works have confirmed the connection between EEG frequency and mobility somatosensory potential (12–15 HZ)^13–16^. Modulation of PFC activity has positive effects on brain functions such as gait^17^, working memory^18^, and specific executive functions (attention, reward, and volitional motricity)^18–20^. By describing the distinct patterns of connectivity of each region, it is possible to better understand the relevant aspects of functional specialization of PFC regions, and in particular, those aspects that are have differential selectivity of their neural populations to specific sensory inputs^4^. Neuronal communication in a set of circuits between basal ganglia and cortical regions through excitatory and inhibitory processes, (basal ganglia and amygdala) is recognized as essential for the disruption of pre-pulse inhibition^21^. Furthermore, inhibition of amygdaloid signaling is involved in emotional learning and retention of fear association^22^ and executive functions dysregulation. Such signaling can be modulated by specific frequencies^23,24^. Previous research suggests that prefrontal cortex ensemble activity and oscillations can be modified with frequency oscillations^25^.

In ADHD, weaker function and structure in PFC circuits were previously reported^26^. MRI studies indicate that volumetric variance in prefrontal/striatal systems and caudate nucleus predicts the severity of parent-reported ADHD diagnostic behaviors^27^.

To date, there are no functionally sustainable modalities of regulating PFC activation levels using a non-chemical, non-invasive, endogenous, self-generating prolonged stimulus in individuals diagnosed with ADHD. Here, we developed and validated a novel neuro-technological system that enables self-generated sustained attention, and increases focus and short-term memory in individuals with ADHD. To this end, we investigated the effectiveness of systematic non-invasive Frequency Specific Cognitive Training (FSCT) with a wireless brain–computer interface (BCI) (headset) during 13 weeks in  a cohort of ADHD subjects. We investigate whether we could detect any improvement in executive functions and working memory, and alleviation of ADHD symptoms. This study aimed to demonstrate that focus and executive functioning can be meliorated by non-invasive training with FSCT.

We hypothesized that ADHD patients will demonstrate altered brain functional patterns following frequency-specific training and that subjects who trained with FSCT would show quantifiable behavioral improvement, as reflected by neuropsychological tests. Additionally, in an exploratory pilot functional MRI (fMRI), we aimed to characterize altered activation patterns in PFC regions during response inhibition conditions, which can be seen in the Supplementary Information section of this manuscript.

## Results

### EEG headset and neuro-program development and validation

**Figure 1:**
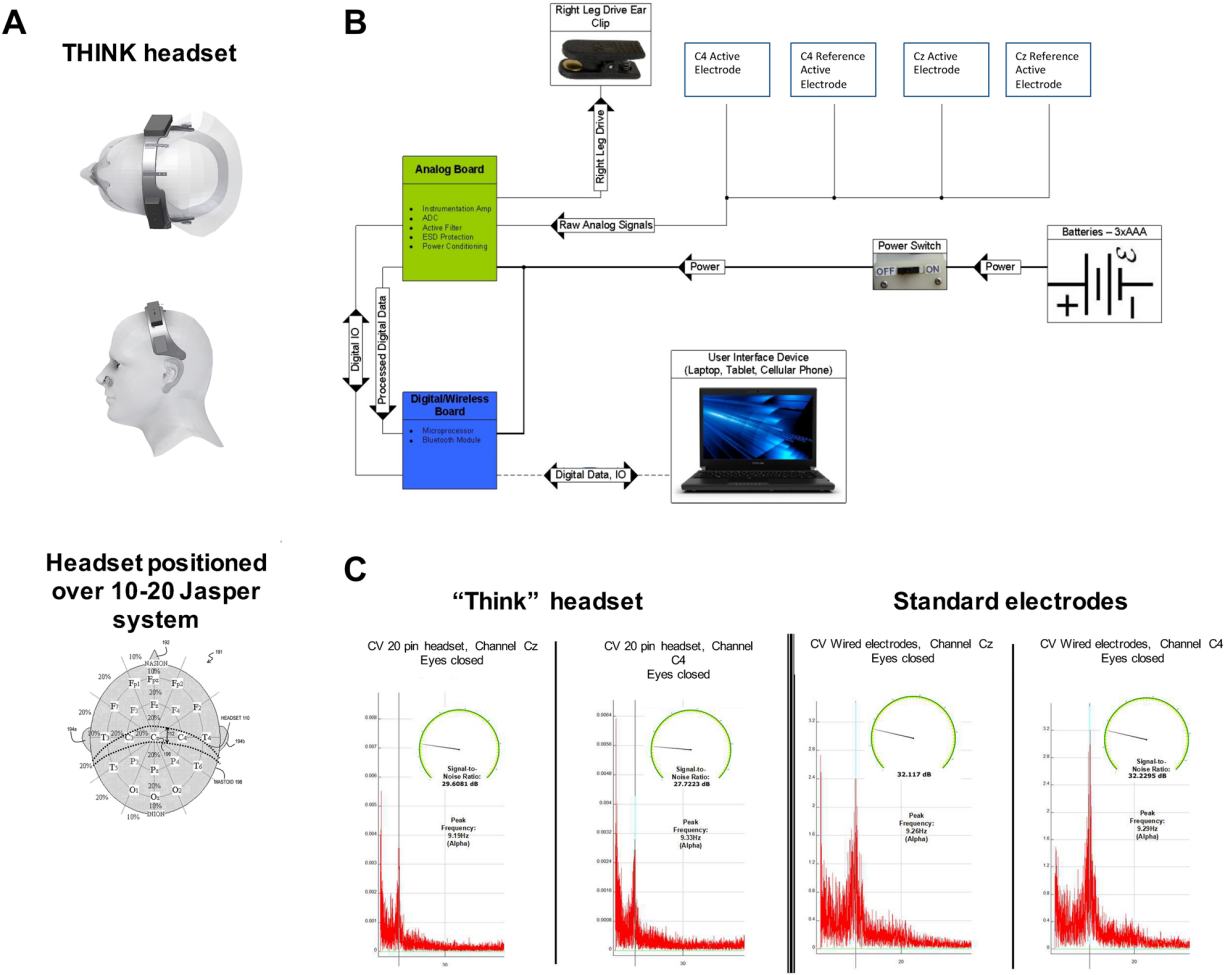
device presentation, positioning, chip and signal validation. (**A**) THINK headset as positioned on a human head, with a reference to the international 10–20 Jasper system. (**B**) System block diagram, showing the integration of analog board and digital process control block. The chip sending the signal to the analog board is encased in the headset shown in panel (**A**). (**C**) Comparison of the signal at Cz and C4 electrode location between THINK headset and Wired Ag/AgCl electrodes, of eyes closed, showing alpha frequencies (9.22 Hz) and signal to noise ratio.

The headset was positioned on a human head, with a reference to the international 10–20 Jasper system with the positioning of the active electrode Cz and C4 at specific locations (Fig. 1A). Two signals were measured; one from the Cz, and another from the C4 region of the skull. These signals were independently referenced from non-active regions of the skull behind the subject’s ear. In this context, an analog system block receives the signal from two active primary electrodes (C4, Cz) and two active reference electrodes (C4, Cz) (Fig. 1B, Supplementary Figures S3–S15), plus the non-active region (behind the ear) and sends it to a digital process control block, which transmits the signal via a Bluetooth wireless protocol to a user interface device (Fig. 1B).

We performed the tests to validate the signals produced by the proprietary electrodes and compared them to traditional Ag/AgCl2 electrodes (Fig. 1C) The BCI Think Headset showed a reliable signal in two independent testing (Fig. 1C and Supplementary Figures S3–S15). Detailed results and statistical data of the comparative tests are provided in the Supplementary Materials.

The headset was functionally coupled with the neuro-program which provided visual stimuli in response to the subject’s reaction to the inputs from the screen. Technical details of the neuro-program are presented in the “Methods” and Supplementary Materials sections.

### Neuropsychological performance

**Figure 2:**
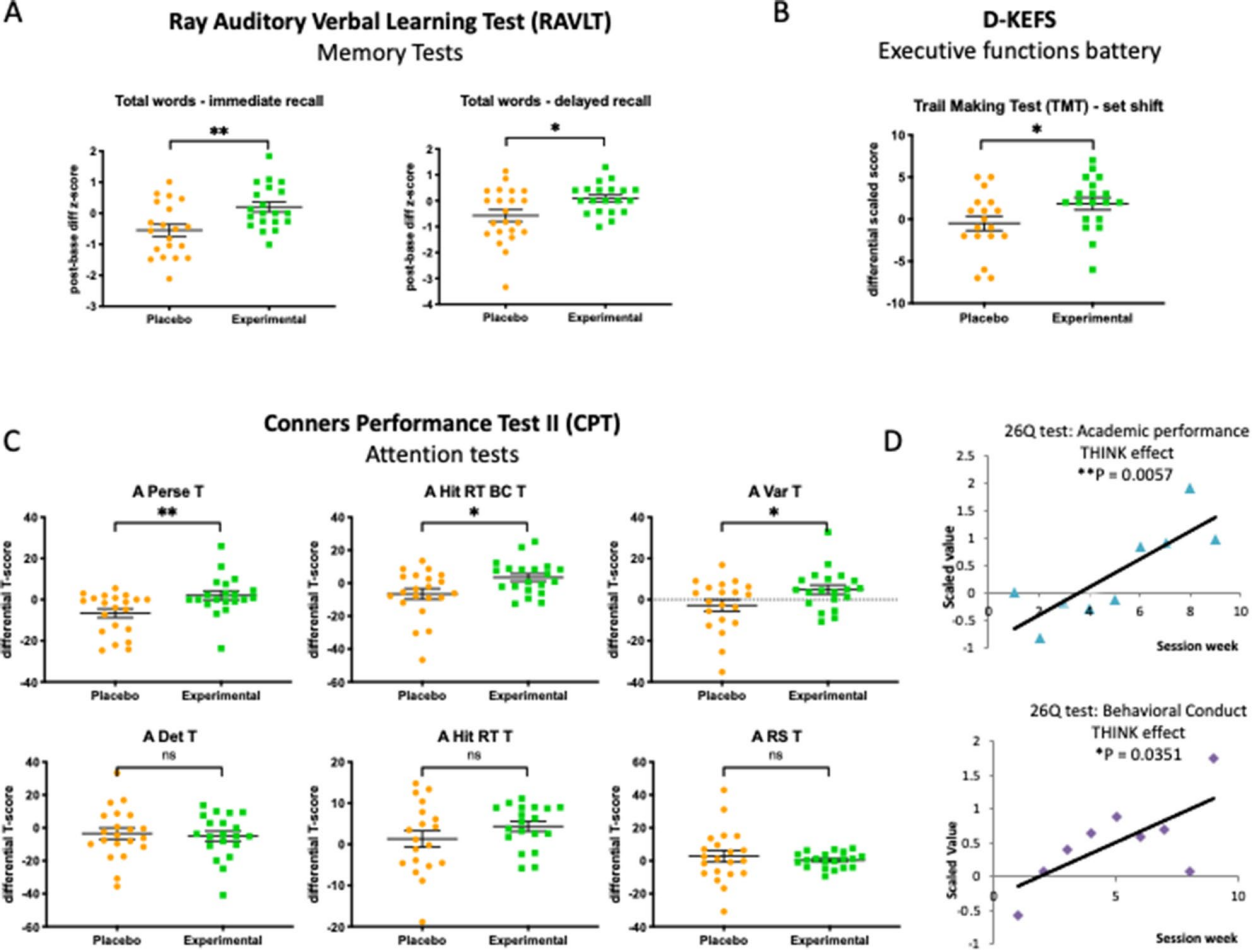
Results from neuropsychological tests, computerized tests and performance questionnaires. 41 subjects (21 in the placebo group and 20 in the experimental group) completed a panel of neuropsychological tests to evaluate global cognitive functioning, attention and executive functions before and after FSCT. Differential scores (post-FSCT vs. pre-FSCT) were calculated for (**A**) Ray Auditory Verbal Memory Test (RAVLT) immediate and delayed recall (differential z-scores normalized by age and gender of the subject), (**B**) Delis–Kaplan Executive Function Test (D–KEFS) Trail Making Test part 4 (TMT-4) *p = 0.0436, (**C**) Conners Performance Test (CPT II) (Unpaired t-test, *p < 0.05 **p < 0.01). (**D**) 26Q questionnaire concerning academic performance (upper panel) and behavioral conduct (lower panel). For each measure, the “THINK effect” was calculated as a subtraction of the arithmetic mean of the results of the experimental group B minus the arithmetic mean of the results of the placebo group A), and its value is shown for each session week along 9 weeks of observation (linear regression, *p < 0.05 **p < 0.01).

*Ray auditory verbal memory test (RAVLT)*. Results from RAVLT show significant improvement in word list learning (for total words recall and delayed recall after 20 min) in the experimental group compared to the placebo group (Fig. 2A). The performance increase of the experimental group was 7.73% of the standardized norms for total words recalled and 20.17% of the standardized norms for delayed recall^28^ versus the placebo one. For immediate recall total words, accounting for sex and age, z-scores were calculated. The experimental group displayed a significant increase in performance (− 0.548 and + 0.2012; *p* = 0.0047 in the experimental group vs. the experimental group respectively). Similarly, in the delayed recall test a significant increase in performance was observed for sex and age group-matched subjects (before vs. z) (differential z-score in the placebo group = − 0.572, while = + 0.0912 in the experimental group), (*p* = 0.017). Therefore, subjects who trained their executive functions with FSCT improved both immediate and delayed memory for words list learning and were able to recall them after 20 min of time lag significantly better.

*Delis–Kaplan executive function system (D–KEFS)*. On a D–KEFS trail-making test, part 4 (TMT-4), the obtained results showed a significant improvement in mental flexibility and set-shift for subjects in the experimental group after FSCT training (Fig. 2B). In TMT-4, the experimental group performed significantly better than the placebo group (*p* = 0.0436). No significant differences were detected between both groups in TMT-1, TMT-2, TMT-3, TMT-5 (*p* > 0.05, in all cases).

The *Conners Computerized Performance test II (CPT II)* was chosen to assess attention-related problems in a task-oriented manner. Significant improvement was observed on CPT-II in the FSCT versus the placebo group for perseveration errors decrease (*p* = 0.0067), hit rate (*p* = 0.015), and accuracy (Var T) (*p* = 0.039) (Fig. 2C).

*26Q Questionnaire*. The 26Q questionnaire was administered to the parents of participants on a bi-weekly basis during the study. The questions measured scholastic grades and behavioral conduct over 9 weeks. When comparing both study groups, the experimental group showed significant improvement for both academic performance and behavioral conduct (*p* = 0.0057 and *p* = 0.0351, respectively) (Fig. 2D).

### EEG results

**Figure 3:**
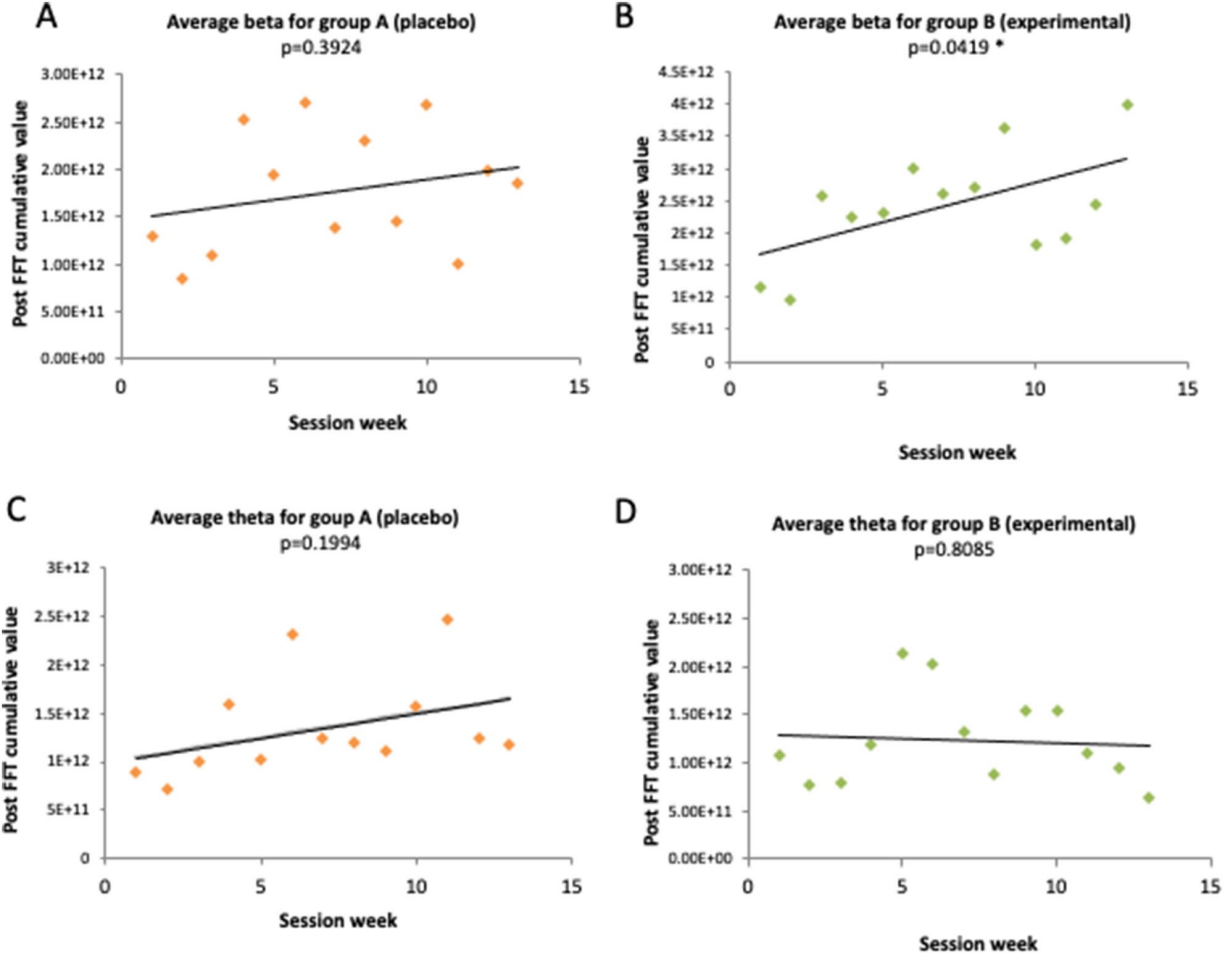
Cumulative average EEG values for placebo group A and experimental group B. EEG frequencies from 21 FSCT (experimental) subjects and 19 placebo FSCT (pFSCT) subjects were collected over a time interval of 13 consecutive weeks. The plots show the average cumulative beta frequencies (which reflect focusing ability and are linked to frontal lobes activity) (**A** and **B**, upper panels) and theta frequencies (which are reflective of distractibility) (**C** and **D**, lower panels) from these subjects. Linear regressions were calculated to evaluate the significance of the trends (*p < 0.05).

The EEG data were collected from all participants at each session throughout the study, annotated and recorded by subject and by session for the entire duration of the session in real-time. EEG results were plotted over 13 sessions and showed an increase of beta frontal activity in those subjects who underwent FSCT (Fig. 3B), but not in the placebo group (Fig. 3A. In the experimental group, beta frequencies showed a significant increase throughout the training, which associates with a higher degree of attention. In particular, the linear regression for the post FSCT cumulative value of the average beta EEG frequency for the experimental group showed an increment in cumulative beta EEG values with statistical significance (*p* = 0.042). In contrast, the same analysis yielded no significance in the placebo group (*p* = 0.392) (Fig. 3A). For the Post FFT cumulative value of the average theta EEG frequency, no significant increase was observed (*p* = 0.1994 for placebo group A and *p* = 0.8085 for experimental group B) (Fig. 3C,D).

### Magnetic resonance imaging (MRI)—see supplementary information

A subset of subjects from each group—experimental and placebo—underwent on fMRI and MRI scan screening before and after FSCT (7 subjects in each group, for a total of 14 subjects).

## Discussion

The neural mechanisms underlying non-invasive frequency-specific training systems in ADHD rehabilitation are still largely underexplored. We set to investigate a possible increase in focus and executive ability in 46 school-age children with ADHD. We created the Frequency Specific Cognitive Training (FSCT), portable electroencephalography (EEG)-based wireless system consisting of a novel headset, electrodes, and neuro-program.

Currently, there is no available cure for ADHD, and symptoms are managed through medications and behavioral therapy. In the present study, we aimed to address an unmet need for an alternative, non-invasive method that can help in the management of ADHD, providing tangible improvement using FSCT training. In this randomized, double-blind, controlled study we observed increased cortical activation in EEG and behavioral tasks performance improved following FSCT training in subjects with ADHD. In the supplementary section, we provide the preliminary data of our pilot study which includes fMRI data in go/no go task.

Over the past 10 years, we developed and validated a novel FSCT brain–computer interface system that targets brain areas implicated in executive functions and attention. This system is composed of a non-invasive BCI, a wireless, portable neuro-technological device, and a program that records the brains' electrical activity in the prefrontal cortex and transmits the information to computer software. The computer software then gives visual feedback via a computer game, triggering a self-induced reward stimulation during the training, where subjects learn to modulate specific brain frequencies.

Based on the obtained results, non-invasive FSCT induced clinically-relevant benefits in several cognitive aspects in school-age children with ADHD. Specifically, we here demonstrate that following training with FSCT, EEG waves are impacted by favoring Beta frequency activity (12–15 Hz) instead of Theta (4–7 Hz) frequency in fronto-dorso-lateral areas in these individuals. Based on neuropsychological and academic outcome measures, FSCT was found to affect executive cognitive functions and ameliorates focusing, working memory, and attention. Previous studies reported changes in PFC region and dopamine receptors activation and anatomical connectivity as reflected by certain EEG frequencies^6^. Patterns of selectivity and connectivity suggested that the posterior lateral PFC (plPFC) contained two segregated regions that belonged to the global dorsal and the ventral visual streams^29,30^, which encoded predominantly visual-spatial and object information, respectively. These interactions between PFC and visual cortices related to attention are dynamic and are mediated by the interactivity between those two structures^31^. This was further supported with neuroimaging methods in humans, that demonstrated the role of the PFC in maintaining top-down control over visual selection and encoding behaviorally-relevant stimuli in various tasks^4,10,32–34^. Furthermore, the subjects who trained with FSCT showed to have ameliorated their performance on Conners CPT II HIT RATE BC and the results are consistent with models of sustained attention that involve the interaction of cortical (frontal, temporal, parietal), subcortical (limbic, basal ganglia), and functional systems including the pathways between the basal ganglia, thalamus, and frontal lobes^35^.

The increased activation observed in these areas can be possibly linked to the observed performance improvement in timing and accuracy after training with FSCT. FSCT includes a specific inter stimulus interval (ISI) which is related to increased attention to detail. Timing-related hypo-activity was previously reported to be linked to the left sub-thalamic nucleus and left pallidal activity^36–38^ areas which play a role in interval timing in visuo-spatial perception of objects^39,40^. As such, the sub-thalamic nucleus and pallidum are considered key areas in the temporal and accuracy monitoring of predictive models^41^.

The limitations of the current study include a relatively small overall sample size. Our ongoing efforts are focused on further establishing these observed findings and including the fMRI and CAT scan data in a larger cohort of ADHD population. Furthermore, we plan to explore the benefits of the FSCT in other patient populations such as Parkinson’s disease and traumatic brain injury.

This proposed technology was found to significantly increase the utility of EEG recordings by eliminating the need for wires and conductive gels, as it decreases the amount of preparation time. Most importantly, FSCT was found to facilitate harnessing the brain’s natural potential. This increases task execution speed and accuracy, while improving working memory. Overall, this study shows that a frequency-specific cognitive training system is a safe and efficient way to improve attention and memory abilities in ADHD school-age children.

## Methods

### Brain-computer interface design and validation: THINK headset, EEG electrodes and system

A wireless neurotechnology device that is operated without hands (hands-free BCI)^42^ and is used to record, digitize and transmit low-noise EEG signals from human subjects was developed. This system is a combination of a hands-free headset, containing 4 electrodes, and a neuro-program (Fig. 1A). The BCI Think Headset and several neuro-games were created in collaboration with Honeybee Robotics and Columbia University. We tested and validated the system in three independent laboratories (John Ferrera; Gimenez and Nowak, IIB Sant Pau, Barcelona). Statistical analysis of the time required for the setup with the BCI Think Headset was compared to the time required to glue the Ag/AgCl electrodes on the subjects’ scalp. Furthermore, the resistance (in KOhms) was measured for both setups and statistical difference between the KOhms values was compared. The headset was positioned on a human head, with a reference to the international 10–20 Jasper system with a positioning of the active electrode Cz and C4 at specific locations. Real-time continuous recording EEG data were collected from each participant at each session throughout the study, recorded by subject and session for the duration of the session. Detailed description can be found in the Supplementary Materials.

### Study design

A controlled, randomized double-blind, two-arm study was conducted at one clinical site (Hospital de Santa Creu i Sant Pau, Barcelona, Spain) to evaluate the effects of FSCT training on executive functions of children affected by ADHD. The study included children and adolescents (6–18 years) diagnosed with attention deficit and/or hyperactivity (attention, hyperactive-impulsive type, combined type). After the selection phase, the subjects were randomly assigned to two arms: the control arm received a placebo FSCT training, while the experimental arm received the executive reinforcement FSCT training. The study lasted for 13 continuous weeks, where executive functions were measured through batteries of neuropsychological tests. ADHD symptoms were rated before and after the training. Furthermore, EEG measurements were evaluated continuously throughout the 13 weeks of training. In addition, data of task fMRI datasets were collected at specific time points from a subgroup of subjects, here included in the Supplementary section.

### Participants selection and recruitment

The research on the investigational, non-invasive medical device developed in this study was conducted following approval of the Clinical Research Ethics Committee at the Hospital de la Santa Creu i Sant Pau (Barcelona, Spain) (EC/11/218/3407), in full compliance with the Medical Device Directive (2007/47/EC) and all relevant regulations.

We screened N = 120 healthy students aged between 6 and 18 years old. Written informed consent was obtained from participants age 18 and older. Those under the age of 18 provided a signed consent. Additionally, parents/legal guardians also provided signed informed consent. All subjects were tested by a team of psychologists and neuropsychologists and were assessed using a tests battery. Inclusion criteria were: pediatric age (6–18 years old), ADHD diagnosis (according to DSM-IV-RT, APA 2000), IQ greater than 80, ability to understand the study design and aims, and willingness to participate. Exclusion criteria were: administration of drugs or cognitive stimulation therapy in the 3 months before the beginning of the study, moderate to severe sensory difficulties (hearing or vision), and other significant medical history reported or found (epilepsy, psychiatric history, head trauma, intracranial implants, uncompensated systemic disease). After the screening visit, N = 41 subjects were enrolled to the study.

Randomization was achieved by a software (www.randomizer.org) into two groups. The experimental group (n = 21) received “Think training” (FSCT) three times weekly for 40 min each, over 13 sessions, with an interval of at least 24 h between sessions. The placebo group (n = 20) received the same treatment, except that the training was a neuro-game with randomly assigned reinforcement (non-training version).

### Study Blinding

Study participants and their parents were blinded to the study group they were assigned to. Researchers, statisticians, and MRI specialists were also blinded to group the assignment of the study participants. The participants in both groups were set up in the same fashion, with a working headset placed on their heads, and seated in front of the screen in the same fashion. The participants in the placebo group did not receive active feedback to their emitted EEG signals instead, they were given a control demo neuro-program with random feedback.

### Questions, neuropsychological and cognitive assessment

*The 26Q questionnaire* is a short questionnaire, created by our group. It is composed of 26 questions and was administered to the parents of participants on a bi-weekly basis during the study inquiring regarding the participant’s scholastic grades (based on report cards), and behavioral conduct over the range of 9 weeks. The parents were blinded to what group their child was in (placebo or experimental).

*Ray Auditory Verbal Memory Test (RAVLT)*: The RAVLT was used to evaluate verbal learning and memory, including proactive inhibition, retroactive inhibition, retention, encoding versus retrieval, and subjective organization^43^. The test lasts about 25 min. The tester reads to a participant a list of 15 words across five consecutive trials. The list is read aloud to the participant, and then the participant is immediately asked to recall as many words as he/she remembers. This procedure is repeated for 5 consecutive trials (Trials 1–5 immediate recall). After 20 min, the tester asked the participant to recall the list once again^45^.

*Delis–Kaplan Executive Function System (D–KEFS):* D–KEFS trail-making test, part 4 (TMT-4), was administered. The trail making test is a pencil and paper timed task that measures flexibility of thinking on a visual-motor number-letter sequencing task. The task is timed and the tester records the time of the completion of the task. The discontinue rule limit is 240 s^47^.

The *Conners Computerized Performance test II (CPT II)*: The CPT II is a timed computerized performance test to assess attention-related problems in a task-oriented manner. It is a commonly used instrument by ADHD research and clinical assessments for participants aged 6 or above. The test is administered on a computer. The participant presses the space bar or clicks the mouse button when a letter other than X shows up onscreen. Letters appear on the screen at different time intervals^46^.

### Statistical analysis of scholastic and neuropsychological performance

For all the neuropsychological analyses, we evaluated the “FSCT effect” as a measurement of the increase in performance after cognitive training sessions. This was measured by subtracting the score obtained at baseline (t1) from the score obtained at postline (t2) (FSCT effect = score_t2_ − score_t1_).

For RAVLT, cumulative answers were expressed as z-scores normalized by the age and sex of the subject. Baseline normalized z-scores were then subtracted from postline normalized z-scores for both the placebo and the experimental group subjects. Statistical differences between the placebo and experimental group were calculated using independent-samples t-test. For D–KEFS, TMT scores were calculated based on the number of successfully completed tasks. Baseline scores were then subtracted from postline scores for both the placebo and the experimental group subjects. Statistical differences between both study groups was calculated using independent-samples t-test. For CPT tests, performance was assessed by dedicated software (CPT3, Conners, 2018) measuring the response to visual stimuli under a series of parameters to evaluate attention and response. The Conners CPT 3 presents 360 scored stimuli trials divided into 6 blocks, with 3 sub-blocks each consisting of 20 trials. The cumulative results of each trial were calculated for each subject before and after training. Baseline scores were then subtracted from postline scores for both the placebo and the experimental group subjects. Statistical differences between the placebo and experimental group were calculated using independent-samples t-test.

### Statistical analysis of 26 questions

The analysis was performed as follows: we considered 9 weeks (from the 5th to the 13th week), under the hypothesis that FSCT training effect becomes evident approximately after 5 weeks. The scores were valued as YES = 1, NO = 0. Each week, we computed the arithmetic mean of the answers’ scores of subjects in the experimental group and of the subjects in the placebo group. Subsequently, we subtracted the latter from the former (experimental-placebo). The resulting values are defined as the “Think effect” on a weekly basis. This produced two-time series, which described the weekly gap between the average score of the experimental group and the placebo group for the two tests. We then computed the linear regression along the 9 weeks for these values and the associated p-value.

### FSCT training

The subjects were sat at one meter distance from the computer screen. The headset was placed on their scalp. They were instructed to sit and watch the program on the screen, paying attention to the motion of the ramp while focusing on the cursor. Their focused state allowed the ramp to lift and produce multiple feedbacks on the computer (scores). Each sitting consisted in 20 trials lasting about 110 s each.

### Neuro-program design

The signal from the headset, transmitted via Bluetooth into the computer, serves as a data output regulator and modifies multiple parameters, such as bubble signal with the score, ramp movements, flying branches, and clouds movements on the screen in real-time, giving the subjects instant real-time feedback on their frequency output.

Participants were seated in a comfortable chair in a dimly lit and electrically shielded room, facing a monitor placed at a distance of approximately one meter from their eyes. Stimuli were presented on a 23″ SAMSUNG screen (1680 × 1050 px) using Unity (Unity Technologies, San Francisco, CA, USA) The EEG signature that is being fed back to the participant is performed in a data-driven manner, our Headset BCI aiming at the control of specific elements of the screen (the ramp). We aim at an EEG profile that becomes more similar to healthy subjects in peak performance state. We calculated the values relative to a baseline measurement and a common baseline was used as a constant across all training sessions, which lasted 30 min. The activity in a specific frequency band (alpha and low beta) was monitored and their ratio was extrapolated (theta/beta training, with a focus on down-regulating theta and enhancing the beta. A reward signal is given when the brain activity is in the desired beta range, with additional positive feedback given when that range is sustained by 700–1000 ms. Positive feedback is associated with the higher score above the bubble, in the foveal region (the score is on a scale ranging between 2 and 20). In addition, negative feedback when brain activity changes in the direction opposite to the intended one is given in a form of a lower score^44^.

The video display shows a video of the “character” on the screen moving in a straight line and passing through the 4 mm diameter white bubbles, positioned in the center of the screen, in the foveal range of the participant. The inter-stimulus-intervals ISIs of the “bubbles and points” feedback is within a randomized range of 400–700 ms; contrast sensitivity of the stimuli on the screen was set at 40% for the stimuli in the foveal region. All stimulus colors on the neuro-program were approximately isoluminant with the background (48.7–60.77 cd/m^2^, luminance background: 57.77 cd/m^2^).

### Electroencephalography (EEG) acquisition

Real-time continuous recording EEG data were collected from each participant at *each session* throughout the study, annotated and recorded by subject and session for the entire duration of the session.

### Statistical analysis of EEG data

Data analysis was performed under the hypothesis that Think training would increase brain frequencies in the cumulative beta range frequencies generally associated with higher-level cognitive performance. EEG results were plotted over 13 sessions. We analyzed the frequencies beta cumulative and theta cumulative for the experimental group and the placebo group. We considered 21 subjects in the placebo group and 19 subjects in the experimental group. The data included 18 effective training days with at least three trials of neuro-training per day. We decided to include only days with at least three trials of neuro-training per day for consistency. Furthermore, data from the training days 16–18 were not included for subjects’ nonattendance in the month of June. Finally, data of days 10–11 were excluded due to technical errors. For the remaining 13 days, since there was a big oscillation of the frequencies across the same day, we considered the maximum value of the frequencies among the three or more trials per day for every subject (both in placebo group and experimental group, both for frequencies beta cumulative and theta cumulative). Consequently, we averaged with a daily arithmetic mean the values of the 21 subjects in the placebo group and of the 19 subjects in the experimental group. As a result, we obtained a daily average (for 13 days) of the frequencies beta cumulative and theta cumulative for both groups. For each of these four time series we computed the linear regression with the associated *p* values for the statistical relevance of the outcome.

## Supplementary Information

Supplementary Information.
