# Supplementary material for: Functional changes in prefrontal cortex following frequency-specific training
Source: Sci Rep. 2022 Nov 24;12:20316. doi: 10.1038/s41598-022-24088-7 (PMC9700664; doi:10.1038/s41598-022-24088-7)
Supplement: Supplementary file 1 — Supplementary Information. [file 41598_2022_24088_MOESM1_ESM.docx]

**Supplementary Information:** Bach-Morrow et al

**Methods**

***1.Headset***

The headset was developed to read, digitize, and wirelessly transmit electroencephalogram (EEG) voltage signals. Various electrode architectures were developed and tested with varying results. The final version of the headset integrated contact-type active electrodes. The headsets repeatedly demonstrated the ability to capture signals from 7 test subjects. This headset is easy to set up, with a set up time less than a minute and requires no gel for enhanced conductivity (as opposed to classical AgCl electrodes which require a longer set-up and a medical adhesive to remain on the scalp). The BCI Think Headset and several neuro-games were created in collaboration with Honeybee Robotics and Columbia University. We tested and validated the system in three independent laboratories (John Ferrera; Gimenez and Nowak, IIB Sant Pau, Barcelona). Statistical analysis of the time required for the setup with the BCI Think Headset was compared to the time required to glue the Ag/AgCl electrodes on the subjects’ scalp. Furthermore, the resistance (in KOhms) was measured for both setups and statistical difference between the KOhms values was compare for 30 subjects. The headset was positioned on a human head, with a reference to the international 10-20 Jasper system with a positioning of the active electrode Cz and C4 at specific locations. Real-time continuous recording EEG data were collected from each participant at each session throughout the study, recorded by subject and session for the duration of the session.

Two signals were measured; one from the Cz, and another from the C4 region of the skull. (We reference international 10-20 Jasper system.) These were independently referenced from non-active regions of the skull behind the subject’s ear. The comprehensive system block diagram is presented in Figure 1 in the Manuscript.

***2. fMRI activation patterns during Go/ No Go task***

A significant post-training increase of activation in the Go/*No-go* condition was observed for the younger-age experimental group (age 8-12) in the left fusiform gyrus, the left superior occipital gyrus (L SOG), and the left precuneus *p* < 0.05, family-wise error adjusted for cluster size (FWEc). No significant difference in brain activation levels was observed in the age-matched placebo group (Table 1 and Figure 4). In older subjects of the experimental group B (age 13-18), post-training activation increase was observed in the cerebellar tonsil, the right postcentral gyrus (R Post CG), the right insular cortex, the bilateral caudate nucleus, and the middle cingulum (*p* < 0.05, (FWEc). In contrast, no significant overtime increase in brain activation was observed in the placebo group (Table 1)

**Table 1:** Activation patterns in brain regions implicated in motor inhibition in the “No-Go” condition in the experimental group for both selected age groups. No significant differences were observed in the placebo groups.

***MRI data acquisition***

MRI datasets was performed were collected from sub-group of N= 21 subjects (6 receiving placebo and 15 receiving FSCT) from each age group (8-12 or 13-18 years old). MRI data were acquired for all patients using a 3T (Achieva Philips, The Netherlands) MR scanner with 8-channels phased-array head coil. The MRI protocol included: a high-resolution Turbo Field Echo (TFE) 3-D T1-weighted sequence (TR/TE/TI = 13.15/2.28/500 ms; FA = 8°, voxel size = 0.9 × 0.9 × 1 mm^3^, 160 slices, acquisition time = 261s), for both resting-state and task functional MRI (fMRI), 400 volumes using single-shot Fast Field echo-planar imaging (FFEPI) sequence (TR/TE = 2000/60 ms; FA = 90°, slice spacing = 1 mm, voxel size = 1.8 × 1.8 × 3.5 mm^3^, 40 slices, acquisition time = 808 s). MRI data sets were collected in real time both pre- and post- FSCT.

***Response inhibition task fMRI (Go/No Go)***

Conners II CPT (Continuous Performance Test) Go/No Go paradigm fMRI was used. Study participants were instructed to press a button every time that an alphabet letterwas presented on the screen, and to withhold response every time the letter ‘’X’’ appeared ^45^.

***fMRI data processing***

fMRI datasets were preprocessed using SPM12 (https://www.fil.ion.ucl.ac.uk/spm/)

This included the following steps: the first ten volumes were disregarded due to equilibrium effects, slice time correction, re-alignment to the first volume image in order to account for any artifacts due to the head movement, and registration to the T1-weighted anatomical image. Subsequently, T1- images were segmented into grey matter and white matter tissue probability maps and were normalized to MNI space based on SPM’s tissue probability maps^46^. Normalization of functional data was performed by applying the resulting deformation fields to the re-aligned and co-registered functional images. The normalized functional data was smoothed using an 8 mm FWHM kernel. Within-subject activation patterns for the different study conditions were calculated using an event-related general linear model (GLM). To differentiate between activation patterns of both conditions, BOLD signal was modeled based on stimuli onset timings of the performed task. The six head motion parameters were included as covariates to the design matrix.

***Task-fMRI statistical analysis***

Group statistics were performed using SPM. All reported results are at P < 0.01; family-wise adjusted for cluster size (*FWEc)* was adopted for all reported task-fMRI. Post-training changes in task-related functional brain activation were also assessed in the placebo group (Group A) and experimental group (Group B) within each age subgroup using a paired samples t-test and ANOVA. For each condition, the following contrasts were tested: t1 (time point 1) > t2 (time point 2), and t2 > t1.

**
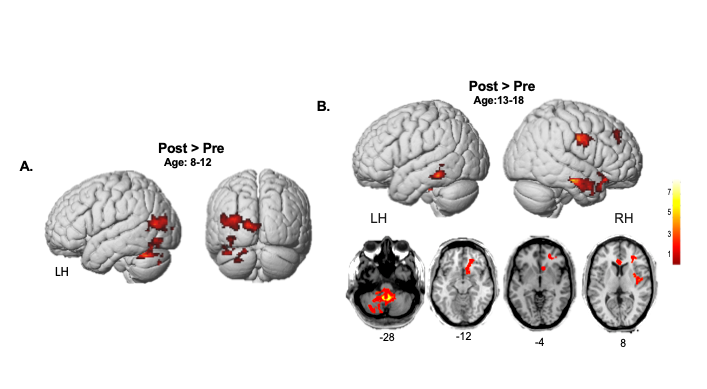
**

**Figure 1 Patterns of post-training significant brain activation increase the “NoGo” fMRI task condition in the experimental groups of both age categories. A)** Subjects aged 8-12 years. In this age group, significant post-training brain activation increase was observed in left fusiform gyrus, the left superior occipital gyrus (L SOG) and the left precuneus. **B)** Subjects aged 13-18 years, significant post-training brain activation was observed in the cerebellar tonsil, the right post central gyrus (R Post CG), the right insular cortex, the bilateral caudate and the middle cingulum (p < 0.05, (FWEc).

## ***3. Analog PCB***

The analog signal processing required for the EEG Headset is typical to that required for many other low amplitude signal monitoring applications (e.g. strain gauge, electrocardiogram (ECG), electronic compass, etc.). A differential signal is input to the system. It is then filtered, amplified, and digitized before being transmitted to the system microcontroller. See Figure 1 in Supplementary data, Analog Circuit Block Diagram.

The positive side of the differential signal originates from typical EEG placement locations (C4 or Cz), and the negative (reference) side originates from a region of the skull with relatively low brain activity (behind one of the subject’s ears). This differential signal is input to an active analog signal processing circuit (AASPC) after passing through an ESD protection stage and passive differential input filter circuit.

The AASPC contains three main stages; instrumentation amplifier (IA), gain, and low pass filter (LPF). A modest gain is applied in the IA stage is followed by a more significant gain in the gain stage. The LPF stage uses an active 4^th^ order Butterworth architecture, and applies a cutoff frequency of about 40Hz. Passive high pass filters (HPF) are placed between each stage to remove any DC offsets. Prior to the first HPF, the raw IA stage signal is output to the ADC. This raw signal is used in determining signal quality, and the level impedance mismatch between the differential signal inputs. The IA stage is also used as a part of the right leg drive (RLD) circuit. The RLD signal is meant to actively cancel out noise signals picked up by the subject’s body, and is output to an ear lobe clip electrode.


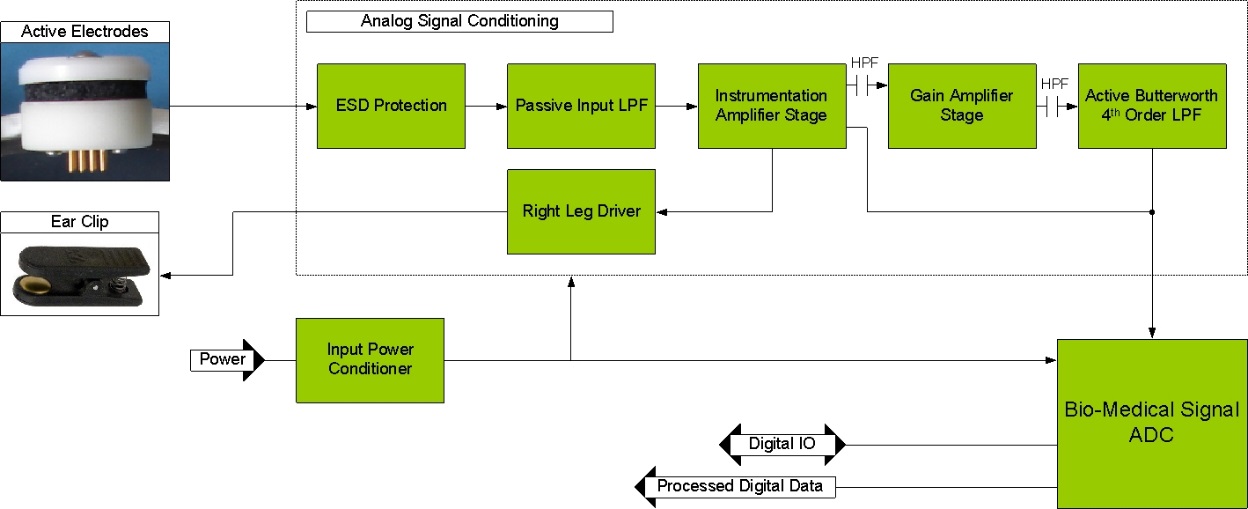


Active electrodes

Ear Clip

**Figure 2:** Analog circuit block diagram

The bio signals are output from the AASPC to a specialized COTS biomedical ADC. The ADC has a high signal resolution of 24 bits and was designed specifically for biopotential signal acquisition. A built in SPI allows the ADC to communicate directly with most microcontrollers. IO (timing, triggering) and data signals are passed between the host microcontroller and the ADC.

Raw battery power is input to the analog PCB and passes through a power conditioning circuit before being distributed to the rest of the board. The input power conditioner was designed to supply low-noise power bus signals to the analog circuit. Positive and negative supply rails are generated using linear regulators.

The power and ADC sections are physically separated from the purely analog section of the analog PCB. The intention was to limit the exposure of the analog traces to noise generating power and digital signals.

## ***4. Active Electrode Module***

There are two main types of electrodes used in biopotential recording devices; Active and Passive. Passive electrodes are typically used in traditional hospital EEG systems and consist of piece of metal (Silver/Silver Chloride) attached to a lead wire. When using passive electrodes for EEG scalp preparation a special conductive paste is required in order to properly capture signals. Passive electrodes were not an option for the EEG Headset design due to the preparation requirements.

Active electrodes (AE) contain active circuitry that precludes the need for scalp preparation. A very high input impedance operational amplifier is used to compensate for impedance mismatch between the two legs of the differential input signal. There are AE designs that do not require skin contact in order to capture a signal. The design used in the EEG Headset, however, required skin contact to function properly. Limited testing was performed with contact free AE designs without success.

The EEG Headset AE uses a unity gain, high input impedance op amp circuit. This circuit was designed into a miniature PCB and is in the immediate proximity of the electrode contact. The electrode contact consists of a pin array populated on a custom PCB. The contact PCB simplifies the assembly of the pin contact array, and is directly mounted to the AE circuit PCB. The pin array is meant to penetrate through the hair of a subject and make direct contact on the scalp. In order to further reduce contact impedance, the pin array is required to be preloaded to the scalp of the subject. The preload strikes a balance between reduced impedance and user comfort. A pliable leaf spring is used to apply this preload. Compliant foam sits between the leaf spring pre-loader and the contact pins in order to act as a passive means for aligning the contact array with the slope of the subject’s scalp. Increased positional adjustment was desired for the reference electrode pair and is accommodated via a swiveling mount.

## ***5. Digital PCB***

The sole function of the digital PCB is to receive digitized biopotential signals from the ADC on the analog board and then transmit the signals via the Bluetooth wireless protocol. Digital filtering options are available within the microprocessor embedded programming and can be enabled or disabled as necessary. The streaming data on the Bluetooth network has a sample rate of approximately 167Hz (6ms period). Four signals are output, and they are all time-synchronized. These signals are the Cz and C4 brain wave signals, and their associated impedance signals. The impedance signals are used to evaluate the integrity of the connection between the electrodes and the subject. Higher integrity connections yield higher quality brain wave data. An impedance signal level of greater than 4E+06 in magnitude indicates a poor connection. (see Fig 2 Supplementary data, Digital PCB Block Diagram)

There are two main components in the digital PCB; the microprocessor and the Bluetooth module. The microprocessor acts as the EEG headset embedded system controller. It actively polls the ADC for data and then sends the data to the Bluetooth module for wireless transmission. The Bluetooth module represents a complete COTS solution for wireless data transmission.

A power conditioning circuit takes the input voltage from the batteries and converts it to a constant digital bus voltage for the digital board components.


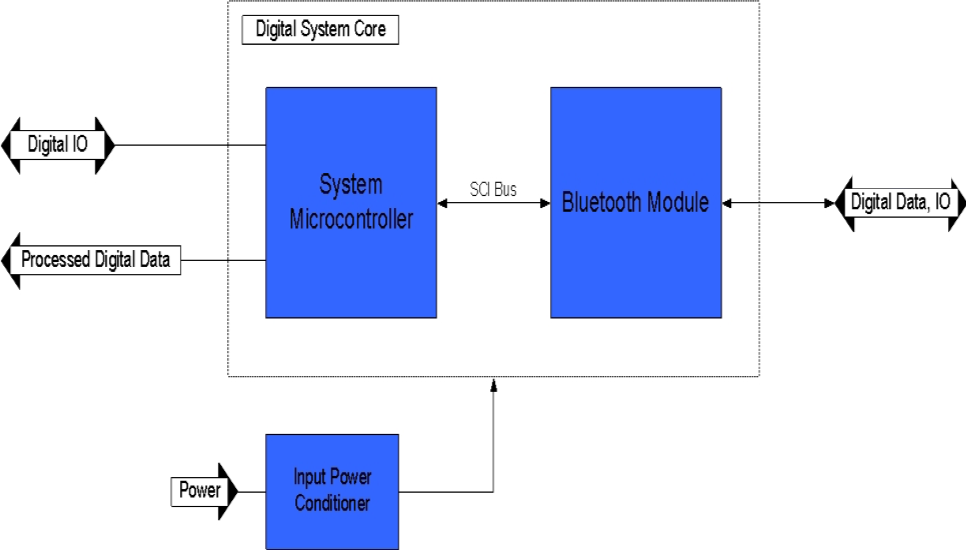


**Figure 3:** Digital PCB Block Diagram

# **Validation and Analysis**

A simple experiment was developed in order to characterize the performance of the EEG Headset prototype system. A test subject was tasked to wear the headset for a set period of time. During the first half of this period, the test subject was told to relax and close their eyes. This activity is known to stimulate the brain into generating the easily identifiable alpha wave pattern. During the second half of the time period, the test subject was given a reading assignment in order to stimulate the generation of beta waves and suppress the alpha and theta waves. Alpha and theta waves are associated with deep relaxation, and light meditation, whereas beta waves are associated with waking consciousness and reasoning. Data was captured during the entire time period. In order to easily distinguish the transition between time periods in post processing analysis, the subject would blink a few times between the first and second halves of the test. The act of blinking would register as a large voltage spike in the time domain data.

Figure 3 (in Supplementary Data), Figure 3: Unprocessed Time Domain Data for Entire Test Period displays the raw time domain data from a typical experiment. The first and second portion of the data is separated by a few large amplitude spikes, and the first half of the data has a greater peak to peak amplitude than the second. This correlates well with the experimental program. Alpha waves generate the greatest peak to peak signal and this is evident in the signal amplitude before the spikes. The spikes represent the subject’s eye blinks, and the last portion of the data represents the expression of beta waves and suppression of alpha waves. More insight is gathered when focusing in on a particular region of data.

3 second windows, from the eyes closed and eyes open (first and second) periods of the experiment were analyzed in greater detail. Figure 4 in Supplementary Data (Figure 4: Time Domain Data from Eyes Closed Period) shows focused time domain data from the eyes open period of the experiment. A clean and almost uniform alpha wave pattern is evident in this data. This is reinforced by the data in Figure 5 in Supplementary Data Figure 5: Frequency Domain Data from Eyes Closed Period, depicting the Fourier Transform of the focused time domain data. Spikes are clearly observed around the center frequency (10Hz) for typical alpha waves, and a very low peak is observed at 60Hz. The 60Hz line frequency is a common source of noise in low level signal monitoring, and the amplitude of the 60Hz spike is a convenient metric for determining the amplitude of the noise present in the measured signal. The signal to noise ratio (SNR) can be estimated by calculating the ratio between the peak alpha spike and the 60Hz noise spike.


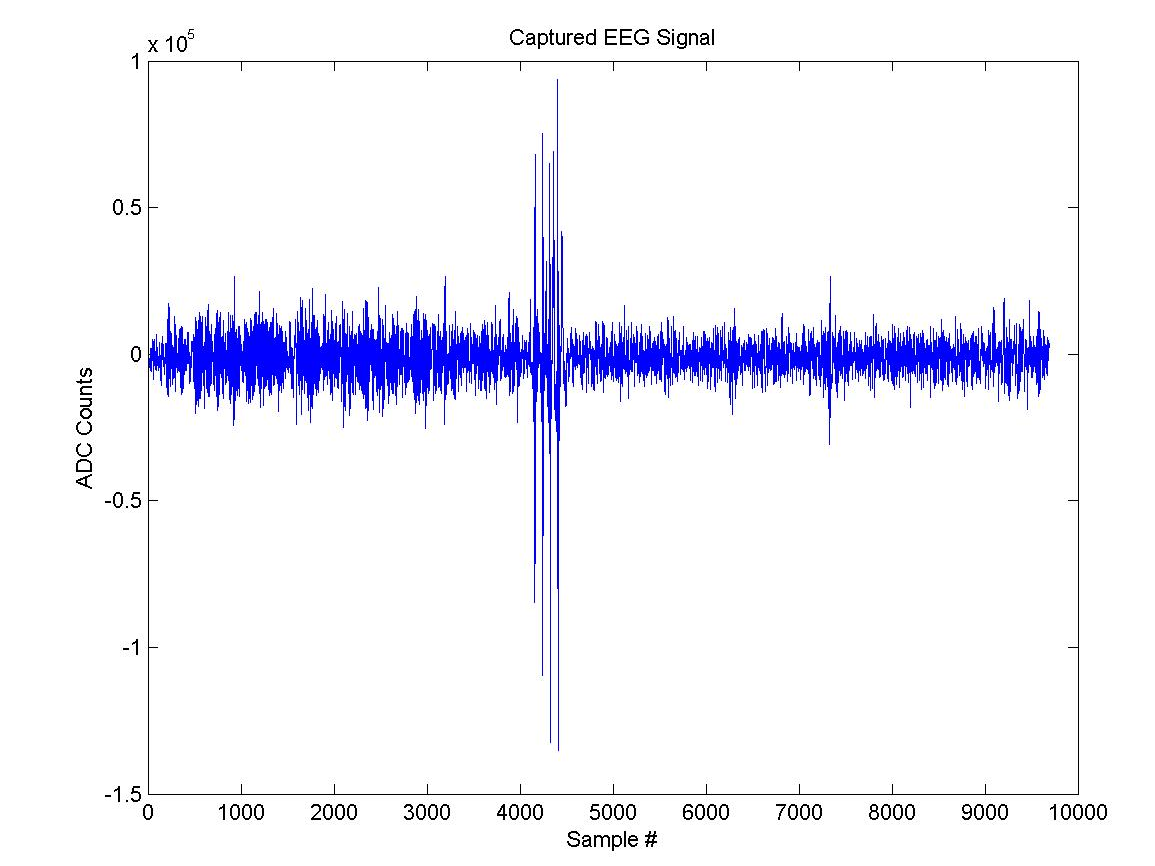


**Figure 4:** Unprocessed Time Domain Data for Entire Test Period


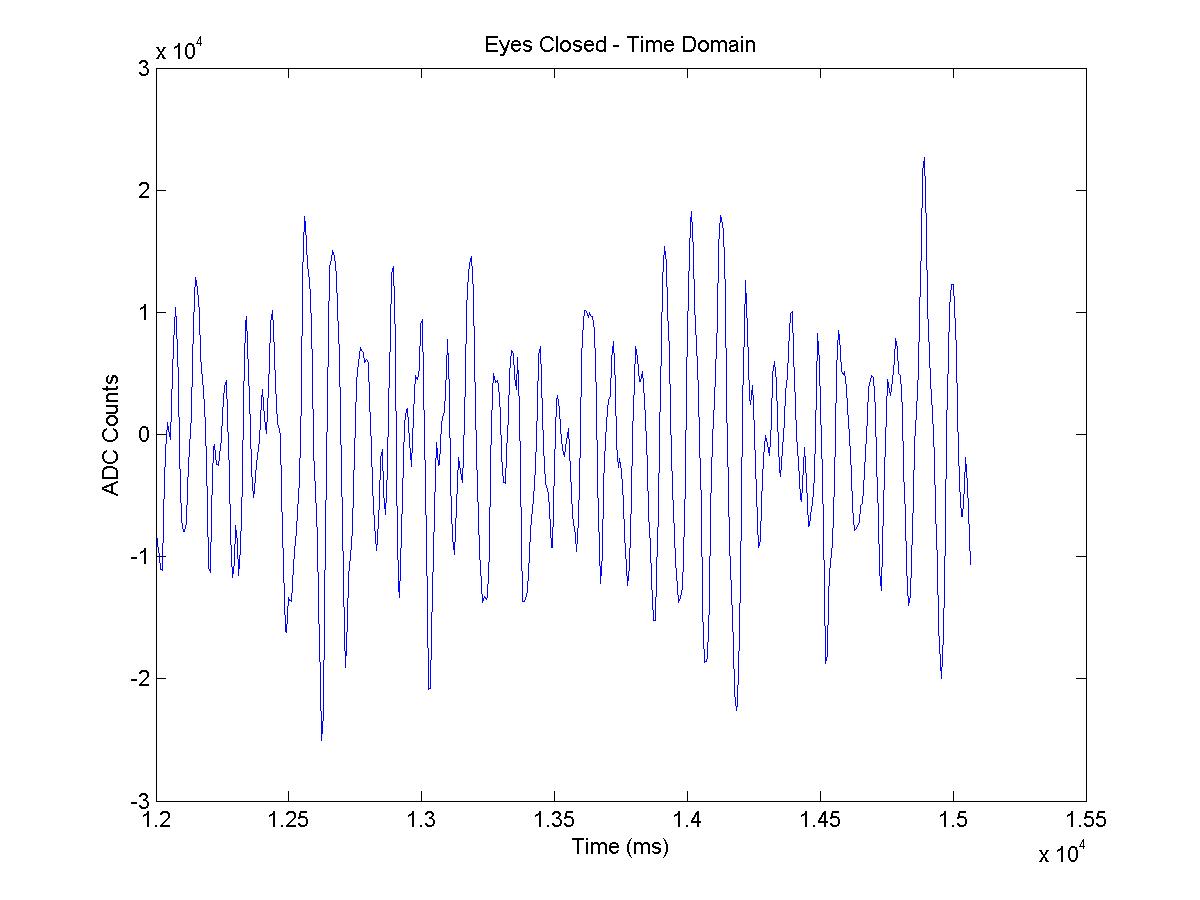


**Figure 5:** Time Domain Data from Eyes Closed Period


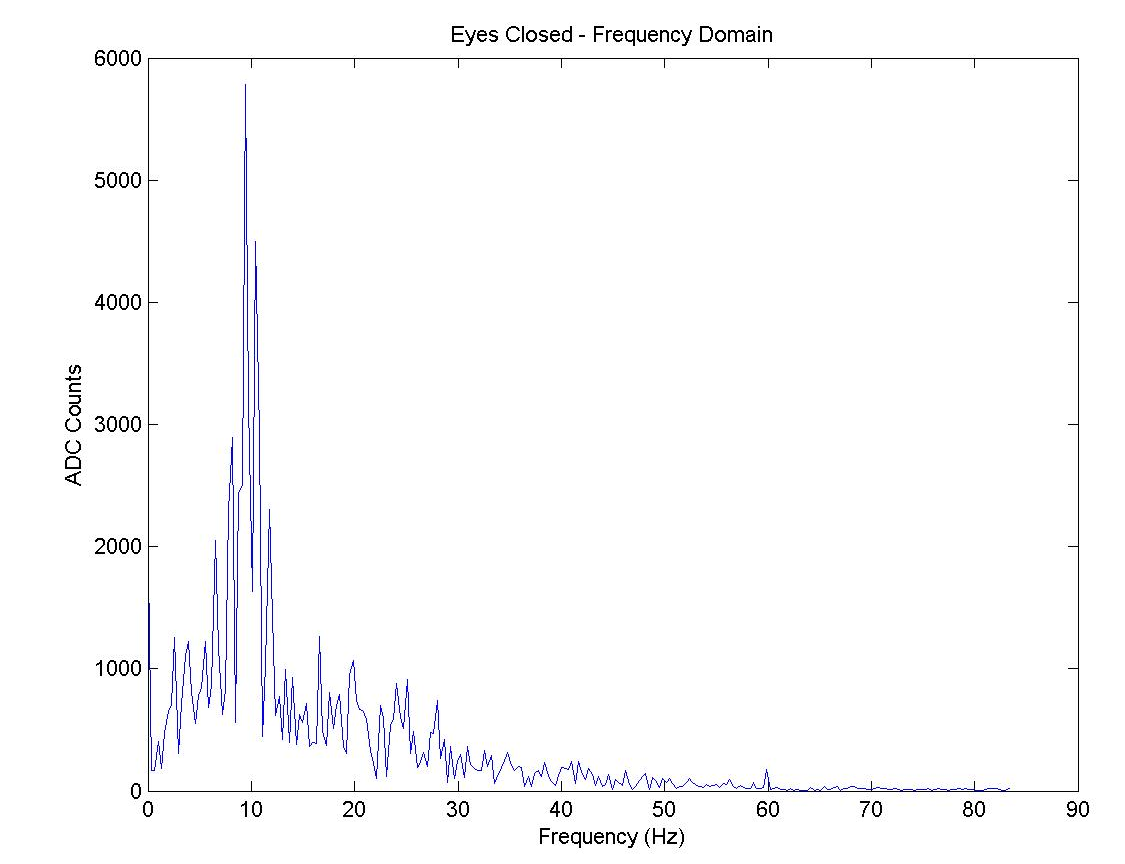


60 Hz Noise Spike

Alpha Wave Spike

**Figure65:** Frequency Domain Data from Eyes Closed Period

Time and frequency domain data for the eyes open period is presented in Figure 6 (Figure 6: Time Domain Data from Eyes Open Period) in the Supplementary Data and Figure 7, respectively. From the presented data, it is evident that the alpha wave signature has been suppressed, and that there is a slight increase in activity in the beta wavelength band (14-40Hz).

After a series of fifty (50) experiments the average estimated SNR was 23.1dB for the eyes closed period and 10.3dB for the eyes open period.


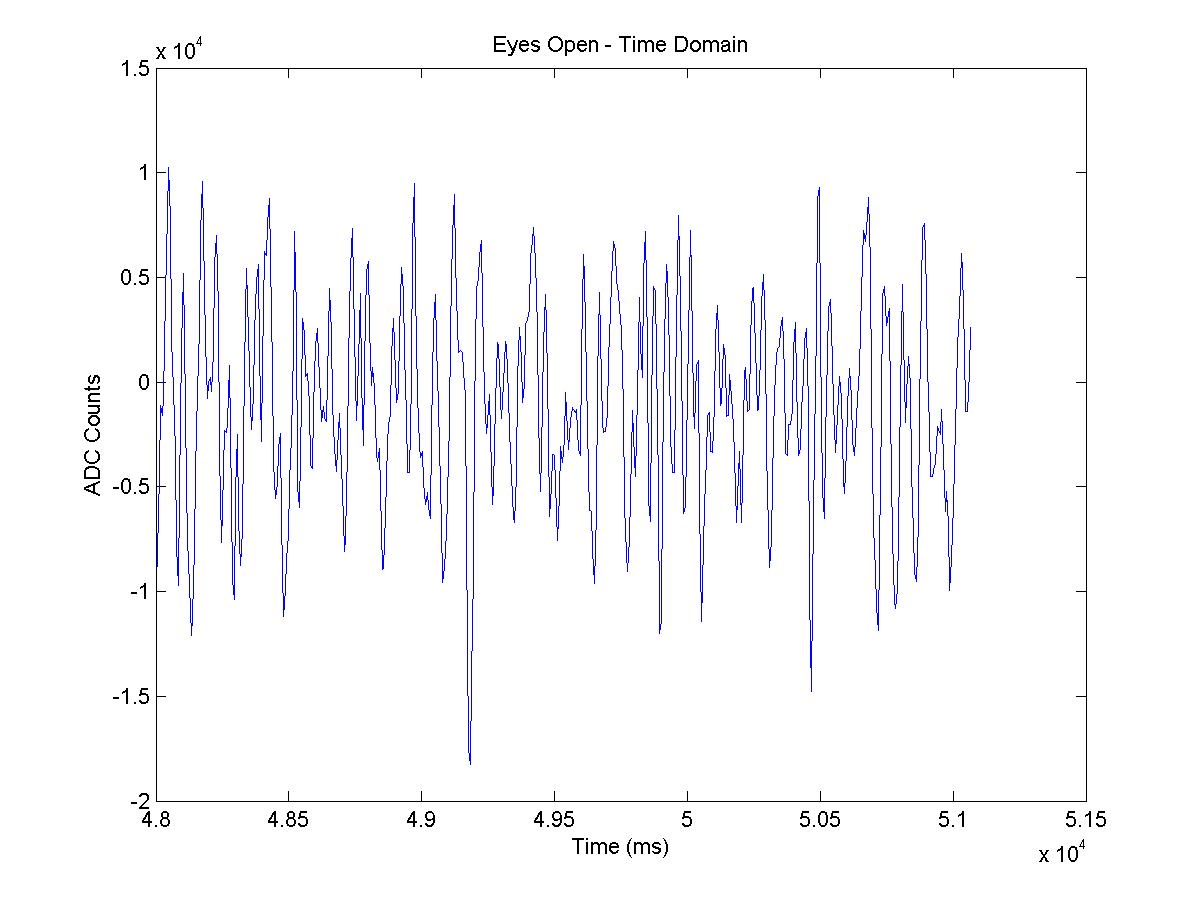


**Figure 7:** Time Domain Data from Eyes Open Period


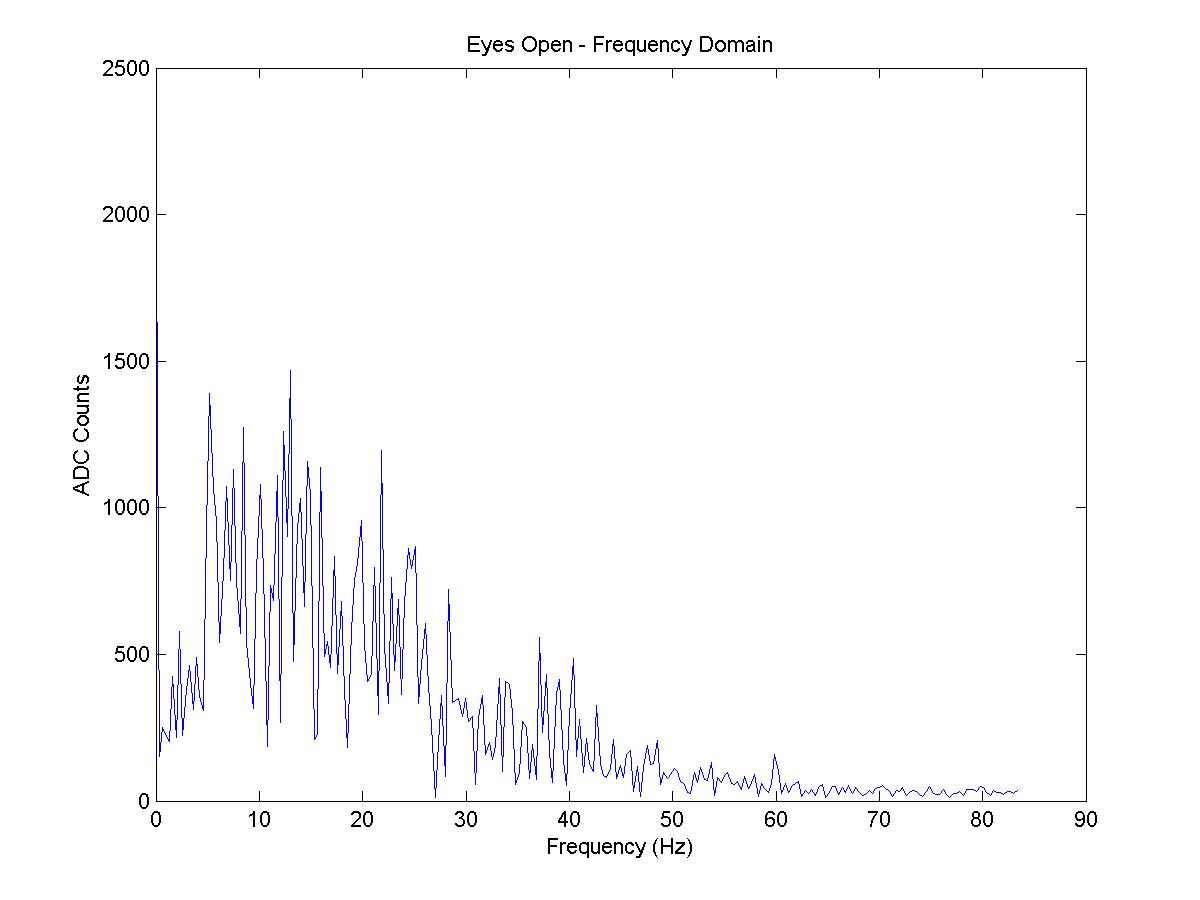


**Figure 8:** Frequency Domain Data from Eyes Open Period

**Validation of the headset by independent laboratories**

***Laboratory 1:***

This validation was performed by a neuropsychologist and EEG practitioner. Trained and working at the Columbia University fMRI Center, where he completed a Master’s thesis and a Ph.D. thesis using various forms of functional brain imaging (i.e. fMRI, DTI and EEG) and brain stimulation (i.e. TMS). He is in a private practice and conducts Neuropsychological evaluations, psychotherapy and academic remediation with children who have learning and developmental disorders, and uses EEG-based frequency training to remediate various disabilities.

A major limitation of all EEG-based frequency training systems is that in order to acquire a clear signal, wired electrodes must be attached to the scalp with conductive paste. While the paste is a very effective means of measuring brain-generated EEG signals from the scalp, it sticks to hair and must be washed out after each recording session. Several dry electrode systems have been created to address this issue, however the signal they collect is not as strong as traditional, wired electrodes. The unique design by this novel headset utilizes a wireless headset equipped with dry electrodes.

In order to test the quality of the signal acquired by the FSCT headset, a quick diagnostic test was conducted. During the diagnostic test, scalp EEG was recorded from the same person using ionized EEG electrodes and the dry electrodes from the FSCT Think headset. The ionized electrodes, called Ag/AgCl2 electrodes, are more sensitive than traditional gold or silver electrodes and thus are capable of recording signals with a higher quality.

Electrodes were placed at two scalp locations, C4 and Cz and were referenced to a linked ears ground (i.e. referential montage). EEG was recorded for a duration of 60 seconds during 4 conditions, Eyes Closed Resting, Eyes Closed Computation (Serial 7's task), Eyes Open Resting and Eyes Open Reading. These 4 functional tasks were chosen because they are known to reliably produce distinct patterns of EEG activity. For the present signal check, we expect that Alpha waves will be more prevalent during the 2 Eyes Closed conditions, and Beta waves will be more prevalent during the 2 Eyes Open conditions. Moreover, we expect the magnitude of the Alpha rhythm to be greater for Eyes Closed Resting as compared to Eyes Closed Computation. Last, we expect the magnitude of the Beta rhythm to be greater for Eyes Open Reading as compared to Eyes Open Resting.

**Comparison By time of setup and the electrode resistance**

We are here providing statistics based on the 30 subjects who were tested with BCI Think headset on, and with the Ag/AGCl were compared by the time to setup the subject and the electrode resistance, in 30 subjects (N=30). The headset was positioned on a human head, with a reference to the international 10-20 Jasper system with a positioning of the active electrode Cz and C4 at specific locations. Real-time continuous recording EEG data were collected from each participant at each session throughout the study, recorded by subject and session for the duration of the session. Statistical analysis of the time required for the setup with the BCI Think Headset was compared to the time required to glue the Ag/AgCl electrodes on the subjects’ scalp. Average time to setup the BCI Think headset was 29 seconds. Average time to setup the Ag/AgCl 4 electrodes was 15 minutes and 33 seconds. The difference of the time of the setup is statistically significant (p=0.00001) It is of note that Think setup does not require scrubbing of the scalp, nor shaving of the hair in a specific spot; it does not require gel or conductive paste, nor the shaving of the scalp from hair on the electrode location. These are significant advantages of the THINK headset setup.

We compared the impedances of the BCI Think electrodes with the resistance of the classical Ag/AgCl electrodes. More specifically, we compared the impedance for electrodes Cz and C4. We found that the BCI Think Cz electrode has a significantly lower impedances as compared to the Cz of the classical Ag/AgCl electrodes (Fig. XA, 2.68 kΩ versus 4.8 kΩ, t 58 = 20.95, p 0.0001). Similar results were obtained for the C4 electrode, that showed significantly lower impedance as compared to the classical Ag/AgCl electrode (Fig. XB, 2.98 kΩ versus 4.98 kΩ, t 58 = 22.73, p 0.0001).

| **Table Analyzed** | **Cz** |
| --- | --- |
|  |  |
| Column B | Ag/AgCl Electrode |
| vs. | vs. |
| Column A | BCI THINK Headset |
|  |  |
| Unpaired t test |  |
| P value | <0.0001 |
| P value summary | **** |
| Significantly different (P < 0.05)? | Yes |
| One- or two-tailed P value? | Two-tailed |
| t, df | t=20.95, df=58 |
|  |  |
| How big is the difference? |  |
| Mean of column A | 2.678 |
| Mean of column B | 4.831 |
| Difference between means (B - A) ± SEM | 2.152 ± 0.1027 |
| 95% confidence interval | 1.947 to 2.358 |
| R squared (eta squared) | 0.8833 |

| **Table Analyzed** | **C4** |
| --- | --- |
|  |  |
| Column B | Ag/AgCl Electrode |
| vs. | vs. |
| Column A | BCI THINK Headset |
|  |  |
| Unpaired t test |  |
| P value | <0.0001 |
| P value summary | **** |
| Significantly different (P < 0.05)? | Yes |
| One- or two-tailed P value? | Two-tailed |
| t, df | t=22.73, df=58 |
|  |  |
| How big is the difference? |  |
| Mean of column A | 2.976 |
| Mean of column B | 4.979 |
| Difference between means (B - A) ± SEM | 2.003 ± 0.08810 |
| 95% confidence interval | 1.826 to 2.179 |
| R squared (eta squared) | 0.8991 |

**Comparison By Task**

In order to measure the *validity* of the acquired signal, EEG data recorded during the 4 different tasks was examined. In particular, data was separated by channel and a frequency analysis was conducted in order to extract commonly used EEG components (i.e. Alpha and Beta). For each channel and within each frequency band, the acquired waveforms from the 4 functional tasks were plotted. The measured pattern activity was inspected to determine if it was consistent with the expected EEG signature. (Figure 8, Supplementary Data)


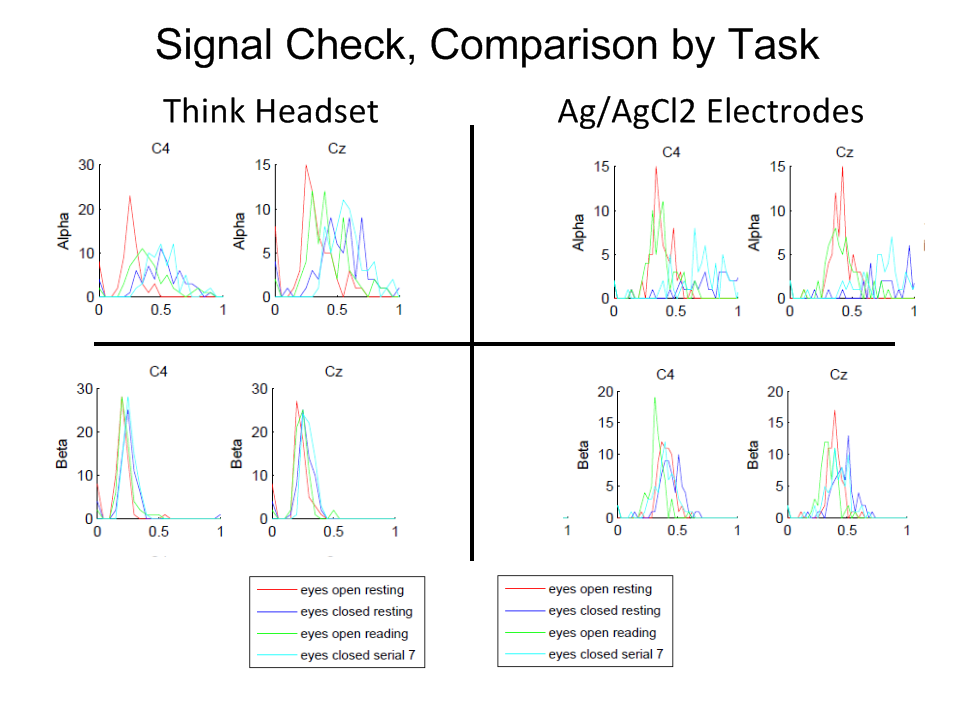


***Figure 9:*** In the top row of two graphs, we present the greatest power in the Alpha band for the Eyes Closed conditions. In the Beta plots on the bottom row of graphs, the separation of frequency bands is not as clear, for both the Think headset and the Ag/AgCl2 electrodes.

**Comparison by Channel**

In order to measure the *strength and quality* of the acquired signal, EEG data recorded from the two scalp sites was compared. Data was separated by functional task and a frequency analysis was used to divide the signal into commonly used EEG components (i.e. Delta, Theta, Alpha, Beta and Gamma). For each task and within each frequency band, the acquired waveforms from each channel were plotted and the amplitude and consistency of the measured signal at each channel was compared. (Figure 9, Supplementary Data)


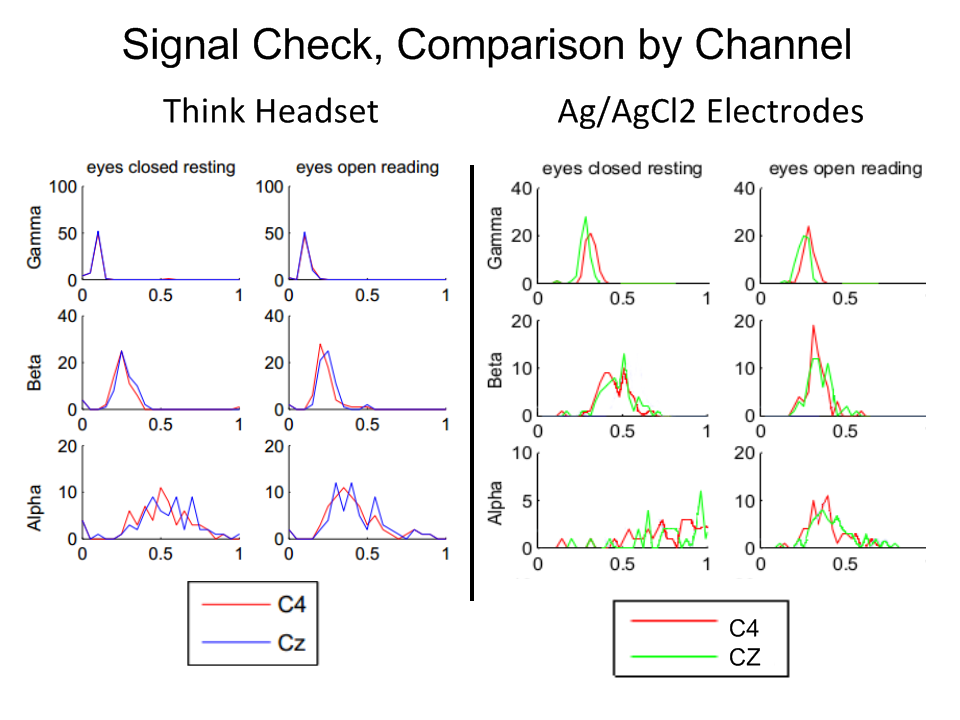


***Figure 10:*** *Comparison of the Signal, by Channel C4 and Cz BCI Think Headset vs Ag/AgCl2 electrodes*

To compare the *strength* of the signal, the magnitude of the waveforms was compared. Results indicate that the signal acquired by the Think headset is at least on par with the signal acquired with Ag/AgCl2 electrodes.

In order to compare the overall *quality* of the signal, the consistency of the signals recorded from C4 and CZ were compared. The consistency of the signal recorded using the Think headset was on par with the consistency of the signal recorded using the Ag/AgCl2 electrodes.

The results of the signal test indicate that the EEG signal acquired by the FSCT Think Headset is comparable to the signal acquired from the traditional electrodes. In particular, the task analysis (Figure 1) indicates that the headset signal is a valid measure of eyes closed and eyes open cognitive states. In addition, comparing results across each channel (Figure 2) indicates that the signal recorded by the FSCT headset is just as strong (and possibly stronger) and less variable than the Ag/AgCl2 electrodes. Results of this quick signal check indicate that the FSCT Think Headset is capable of recording scalp potentials that are on par with traditional EEG recording systems. Not only was the signal found to be a valid measure of scalp EEG, the strength and quality of the signal collected by the two systems was consistent. The results of the present signal test indicate that the Think Headset is capable of recording high-quality EEG signals without using conductive paste.

**Laboratory 2:**

THINK Headset device for acquisition, digitization and wireless transmission of EEG signal, Headset model/serial number 42e2 and 42e5.

Sandra Gimenez MD, PhD and Rafal Nowak, PhD at S Pau and S Creu Hospital, Barcelona A simple experiment was developed in order to characterize the performance of the EEG THINK Headset system. A test subject was asked to wear the headset for a set period of time. During the first half of this period, the test subject was told to relax and close their eyes (EC). This sort of activity is known to stimulate the brain into generating the easily identifiable alpha wave pattern.

During the second half of the time period, the test subject was given a reading assignment in order to stimulate the generation of beta waves and suppress the alpha and theta waves (EO).

Time and frequency domain data for the eyes open (EO) and eyes closed (EC) periods have been analyzed.

Figures (Supplementary data) present results of the analysis, 1. raw time domain data for Cz (Fig 10) and C4 (Fig 11) electrodes; for both conditions (EO – Fig 12. and EC Fig 13,

Figure 14 shows frequency domain data (power spectral density), and Figure 15 shows impedance values over time.

There are clear differences between eyes-closed and eyes-open conditions with evident alfa peak for EC condition and alfa suppression for EO condition.

We conclude that the EEG headset during a test was fully operational and data acquired during the test demonstrated the ability of the tested device to capture EEG neurophysiological signals.


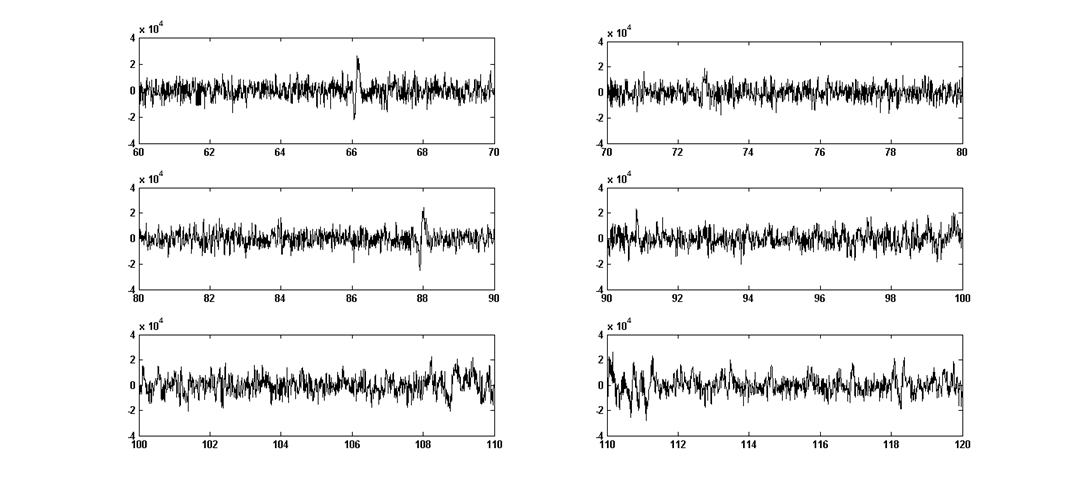


***Figure 11:*** *Bench data Open Eyes C4 Gimenez and Nowak*


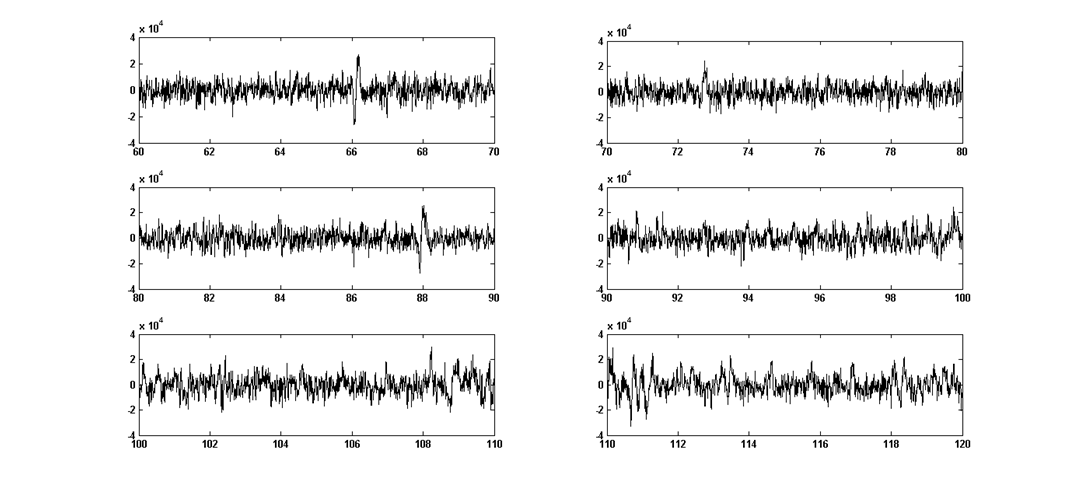


***Figure 12:*** *Bench data Open Eyes Cz Gimenez and Nowak*


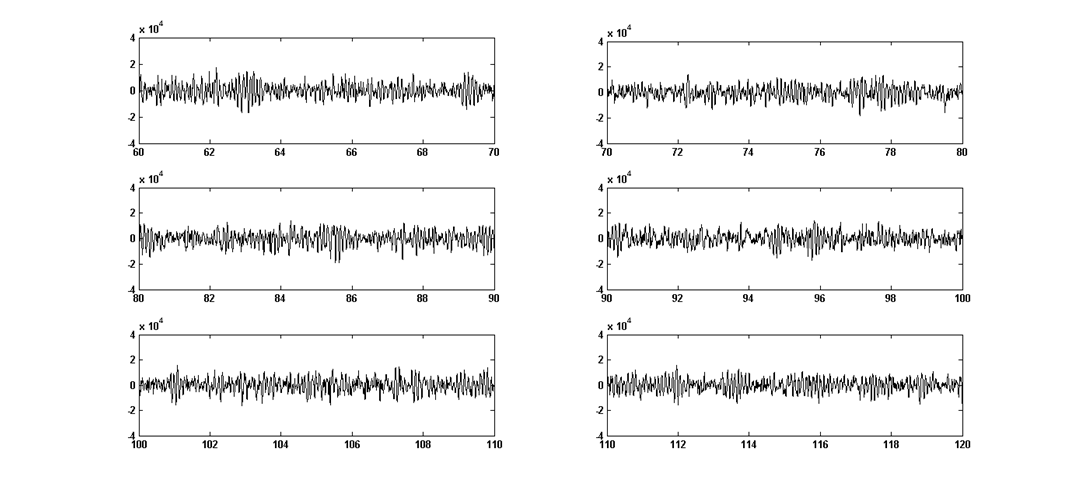


***Figure 13:*** *Bench data Closed Eyes C4 Gimenez and Nowak*


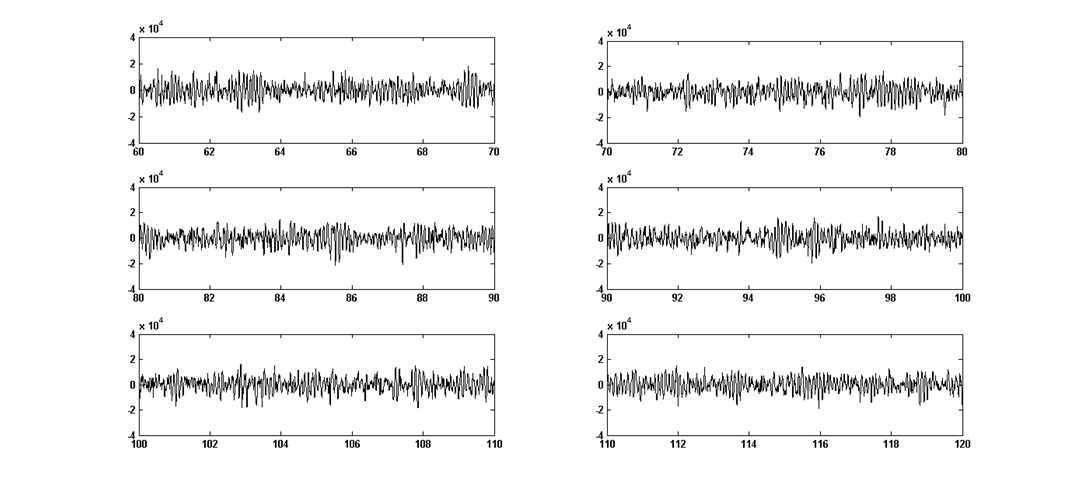


***Figure 14:*** *Supplementary data Bench data Closed Eyes Cz Gimenez and Nowak*

**NOT-NORMALIZED POWER SPECTRAL DENSITY OPEN EYES vs CLOSED EYES THINK DEVICE**


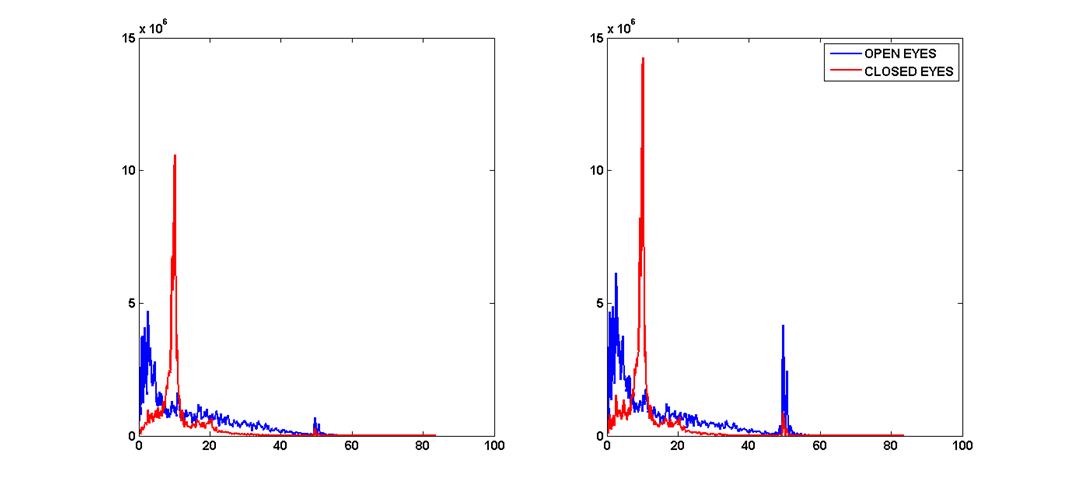


***Figure 15:*** *Frequency domain data (power spectral density) Open and Closed eyes*

**C4 AND Cz ELECTRODES (LOWER THAN 4·10^6^) THINK DEVICE**


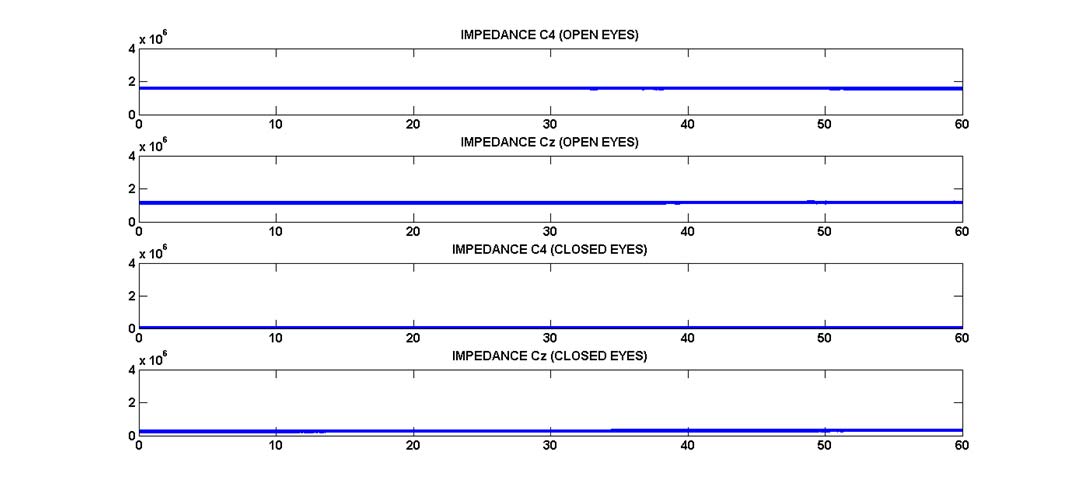


***Figure 16:*** *Impedance values in C4 and Cz, Open and Closed Eyes over 60 seconds time*.

**Neuro-program**

We created a neuro program with the Columbia University department of Architecture (Collins, Morrow, Hasegawa, 2012) to interact with the brain computer interface and train the brain via non-invasive visual frequency feedback loop. The signal from the headset, transmitted via Bluetooth into the computer, serves as data output regulator and modifies multiple parameters, such as ramp movements, flying branches and clouds movements on the screen in real time, giving the subjects an instant feedback on their frequency output. The programs were designed to give Skinnerian feedback to the subject, based on multiple parameters. In creation of the elements of feedback we took in consideration inter stimulus intervals ISIs (all were within randomized range of 400-700 msecs) and specific contrast sensitivity of the stimuli on the screen (40%) especially those in the foveal region, to meliorate and enhance the subject’s response.

The system trains executive cognitive functions and ameliorate focusing, working memory, attention, and mental resilience. No adverse events were recorded from using. A scheme of the system is provided in Figure 1, while details on specific functions are provided as Supplementary Methods.

**FFT and Signal Processing Algorithm**

We keep a buffer of 166 values gathered over 1 second of device signal

These are supplied as signed 3-byte integers.

**Byte Processing Code**

“byteBuf” array contains the 12 bytes (3 x 4 bytes) that describe the signal we receive from the device “intBuf” is an array of the 4 integers these bytes represent

**Fast Fourier Transforms Library**

We use the “JTranforms” library for computing the fast Fourier transform of the signal data in Java. Using Java ensures the Portal will be cross-platform compatible. **JTransforms** is the first, open source, multithreaded FFT library written in pure Java.

<http://sourceforge.net/projects/jtransforms/>

<https://sites.google.com/site/piotrwendykier/software/jtransforms>

This code is derived from General Purpose FFT Package written by Takuya Ooura (http://www.kurims.kyoto-u.ac.jp/~ooura/fft.html) and from JFFTPack written by Baoshe Zhang (<http://jfftpack.sourceforge.net/)>

The library is made available through Parallel Colt “Open Source Libraries for High Performance Scientific and Technical Computing in Java”

Package name “edu.emory.mathcs.jtransforms.fft”

The FFT uses the following function:

Computes 1D Discrete Fourier Transform (DFT) of complex and real, double precision data. The size of the data can be an arbitrary number. This is a parallel implementation of split-radix and mixed-radix algorithms optimized for SMP systems.

Signal Confidence Scores Code

czImpBuffer and c4ImpBuffer contains the 166 impedance values

The goal is to figure out what % of the signal for that second is valid. This % is sent into the game. Currently, the game is very permissive, using any electrode values as long as this “confidence score” is higher than 0.

c4Conf = czConf = 0;

for (int i=0; i<N; i++) {

if ( abs(czImpBuffer[i]) < maxImp) czConf++;

if ( abs(c4ImpBuffer[i]) < maxImp) c4Conf++;

}

c4Conf =ceil( c4Conf/float(N) * 100.0 );

czConf =ceil( czConf/float(N) * 100.0 );

Sending the Signal Into the Game

Before sending the ratios to the game, we:

1. multiply them x4 to get the range of values the game expects (0-20). This range was built into the basic game elements at an early stage of development.

2. “floor” the values to create integers

3. cap any ratios at a predefined max (20) to avoid breaking game mechanics

**Use of the Values in NeuroGame**

NeuroGame elements respond to the following formula (with values of c4 and cz expected to be between 0-20)

int signal = Mathf.RoundToInt( Mathf.Max(1.0f, (SignalManagerRef.c4+SignalManagerRef.cz) / 2.0f) );

In english, we average the signals coming from each electrode, giving always at least a value of 1 (this value is used for scoring, so we are avoiding a score of 0). Game elements (scoring, movement and positioning of elements, and in level 3 the speed of the character) respond to this signal variable.

**“Demo mode” Signal**

Our simulated signal when a device is not connected is created through the following algorithm:

c4 = 20 * Mathf.PerlinNoise( Time.time*.65f,10.0f);

cz = 20 * Mathf.PerlinNoise( Time.time*.65f,20.0f);
